# Caspase Cleavage Sites in the Human Proteome: CaspDB, a Database of Predicted Substrates

**DOI:** 10.1371/journal.pone.0110539

**Published:** 2014-10-17

**Authors:** Sonu Kumar, Bram J. van Raam, Guy S. Salvesen, Piotr Cieplak

**Affiliations:** Sanford Burnham Medical Research Institute, La Jolla, California, United States of America; The University of Texas MD Anderson Cancer Center, United States of America

## Abstract

Caspases are enzymes belonging to a conserved family of **cysteine-dependent aspartic-specific proteases** that are involved in vital cellular processes and play a prominent role in apoptosis and inflammation. Determining all relevant protein substrates of caspases remains a challenging task. Over 1500 caspase substrates have been discovered in the human proteome according to published data and new substrates are discovered on a daily basis. To aid the discovery process we developed a caspase cleavage prediction method using the recently published curated MerCASBA database of experimentally determined caspase substrates and a Random Forest classification method. On both internal and external test sets, the ranking of predicted cleavage positions is superior to all previously developed prediction methods. The *in silico* predicted caspase cleavage positions in human proteins are available from a relational database: CaspDB. Our database provides information about potential cleavage sites in a verified set of all human proteins collected in Uniprot and their orthologs, allowing for tracing of cleavage motif conservation. It also provides information about the positions of disease-annotated single nucleotide polymorphisms, and posttranslational modifications that may modulate the caspase cleaving efficiency.

## Introduction

Caspases are proteolytic enzymes that cleave a limited number of peptide bonds in proteins to regulate their function in diverse biological pathway(s). To date, 11 distinct caspases have been identified in humans with a similar number of homologs in other mammals [1]. They are involved in several functions such as the immune response, DNA replication, cell cycle progression, cell proliferation and apoptosis [2], [3]. The most prominent feature of caspase-specificity is that caspases cleave their substrates almost exclusively after D residues. However, it has also been observed that a E residue at this position could sporadically replace D.

Human caspases are divided into apoptotic (caspase-2, -3, -6, -7, -8, -9, and -10) and inflammatory (caspase-1, -4, and -5) members. The apoptotic members have been further sub-divided into initiators (caspase-2, -8, -9, and -10) and effectors (executioners) (caspase-3, -6, and -7). The initiator caspases have long pro domains containing a death-fold (death effector domain or caspase-recruitment domain (DED or CARD, respectively)) and require forced dimerization in a receptor complex for their activation, whereas the executioner caspases have short pro-domains, exist as dimeric, inactive zymogens in the cytosol and require cleavage by an upstream protease (such as an initiator caspase) for their activation [4]. Based on the analysis of a number of cleavage site characteristics for apoptotic caspases it has been found that the caspase cleavage site has a general motif (DXXD-A/G/S/T), pointing to the overlapping specificity of this family of enzymes [5]–[7]. Thus, caution is required when assigning a cleavage event to an individual caspase based on the cleavage motif alone. Besides the observed specificity for Asp residue at P1 there are other requirements before a peptide or protein can be considered a ‘good’ substrate for a specific caspase [8]. For example, it has been observed that a small and uncharged residue (A, G, S, T and N) is preferred at the P1’ position [9], while residues preferred at P4 are D for caspases-3/-7, I/L for caspases-2/-8/-9/-10, or W/Y, V for caspases-1/-4/-5/-14, -6. At the P3 position all caspases prefer an E residue while no specific amino acid preference exists for the P2 position [10]. The binding site nomenclature is in accordance with Schechter and Berger [11].

In this study, we focus on the *in silico* prediction of human caspases substrates. During apoptosis, caspases initiate, coordinate and accelerate cell death and dismantling by cleaving crucial structural and enzymatic proteins. The cleavage efficiency depends on many factors including posttranslational modifications (PTMs) [12] and may be influenced by Single Nucleotide Polymorphisms (SNPs) that occur near the cleavage sites. Both effects may either increase [13] or decrease the cleavage efficiency, which means that they effectively regulate proteolysis. To understand the importance of the cleavage site motif and its regulation, one should carefully analyze the conservation of such cleavage sites in various organisms [14]. Hence, we designed a substrate prediction algorithm based on amino acid sequence specificity and predicted structural elements, and created CaspDB, a database of predicted caspase cleavage sites in human proteins. Our database integrates information about the cleavage positions with information about the conservation of cleavages in orthologous proteins, and available knowledge about the SNPs and PTMs.

To date, several scoring functions or machine learning techniques have been implemented to predict caspase substrates. For example, GraBCas predicts potential caspase cleavage sites using position specific scoring [15]. PeptideCutter uses a limited experimental dataset to predict cleavage sites for a variety of proteases including some caspases [16]. PoPS provides a set of predefined protease specificity profiles or custom input cleavage site profiles, which are calculated based on the frequency of every amino acid at different positions in the vicinity of the cleavage site [17]. CAT3 is another tool based on scoring matrices, which predicts putative cleavage sites for caspase-3 [18]. CASVM [19] and PCSS [20] are support-vector-machine-based approaches that recognize putative cleavage sites of caspase substrates. Song *et al.* built Cascleave 1.0 using amino acid sequence, secondary structure, solvent accessibility and disorder region features as input to the support vector regression (SVR) models [21]. Recently, its second version, Cascleave 2.0 [22], has been developed for predicting potential cleavage sites for caspase-1,-3,-6,-7,-8. The data for training their SVR model is obtained from the MEROPS [23] and Casbah database [24].

However, existing caspase cleavage prediction methods do not incorporate information about substrate conservation in other model organisms, or PTMs and SNPs that may influence cleavage efficiency. In addition, databases that list experimentally-derived putative caspase cleavage events using mass-spectrometry methods, such as Degrabase, are incomplete because of the low abundance or instability of certain substrates, the absence of other substrates from the cells used in the experiments or because tryptic digest of newly-cleaved proteins yields peptides that are either too small or too large to be accurately detected by MS/MS.

For this study, we designed a novel caspase substrate prediction approach, which illuminates cleavage motif conservation and the influence of PTMs and SNPs. We tested several machine-learning classifiers and compared them with the selected available models. We selected the best model, a Random Forest classification method, and used it to predict potential caspase cleavage sites in all reviewed human proteins from the Uniprot portal [25]. A prediction model was constructed from an experimentally determined caspase cleavage dataset from the MerCASBA database [26], which is a curated version of the Casbah database [24]. All predicted cleavage sites were stored in a database named CaspDB. Due to overlapping cleavage motif selectivity of caspases and the lack of sufficient number of known substrates for some of the caspases, we avoided assigning the predicted cleavages to specific caspases. This assignment could be inferred *a posteriori* by comparing the predicted motif to the commonly observed cleavage sequence for each caspase, as described above. Obviously, our method produces many false-positive hits. This over-prediction is characteristic for all methods for proteolytic substrates prediction, since availability of the substrate and the influence of exosites cannot always be taken into account. However, our method consistently assigns high scores to the experimentally observed cleavage sites, and rank-orders them significantly better than previous methods. Thus, our database should be considered a useful tool to support and guide experimental exploration of pathways involving caspases.

## Results and Discussion

### Evaluation of the classifiers to predict cleavage sites

Caspases are known to have a restricted substrate specificity for an aspartic acid residue at the P1 position while it is less restricted at P4-P2 and P1’ positions in their target sequence. However, due to heterogeneity of the target sequences, the prediction of cleavage sites is a non-trivial task. Four classifiers (Table 1) were trained to predict caspase cleavage sites in protein sequences. Analysis of the training results allowed us to select the best classifier. Because for all classifiers the accuracy and AUC (area under the receiver-operating characteristics curve) have high values, they could not be the sole criteria for assessing classification models. For this purpose, we used several other characteristics including Cost-benefit analysis values and Kappa statistical values [27], analogous to a correlation coefficient. Among the four classifiers, Random Forest was shown to yield highest kappa values, AUC, accuracy, and lowest cost for testing wrongly predicted cleavage sites. We therefore chose Random Forest as the most suitable model for predicting caspase cleavage sites. We applied this method to predict all potential caspase cleavage sites in an annotated set of more than 20,000 human proteins from Uniprot. The results of this prediction are provided by the CaspDB relational database.

**Table 1:** Quality measures of trained classifiers and comparison with publicly available prediction model [44].

	TP	FN	FP	TN	Kappa	AUC	Cost	ACC	PRC	SPC	MCC
RF	191	6	5	788	0.97	0.998	8	98.89	97.0	99.0	0.97
NB	197	0	13	780	0.96	0.999	10	98.69	94.0	98.0	0.96
J48	189	8	4	789	0.96	0.958	12	98.79	98.0	99.0	0.97
SMO	191	6	7	786	0.96	0.998	10	98.69	97.0	99.0	0.96
**RF_combined_set**	644	16	24	2614	0.96	0.999	36	98.79	96.0	99.0	0.96
PeptideCutter								50.8	63.0	97.7	0.05
GraBCas								67.7	67.6	67.5	0.35
CASVM P4-P1								62.3	83.0	93.7	0.32
CASVM P4-P2’								72.7	73.1	73.6	0.45
CASVM P14-P10’								83.1	81.6	80.1	0.66

Abbreviations: TP – number of true positives, FN-false negatives, FP-false positives, TN-true negatives, ACC-accuracy, PRC-precision, SPC-specificity, MCC-Matthews correlation coefficient, Kappa-Kappa statistical value, RF-Random Forest method, NB- Naïve Bayes, J48-decision tree algorithm, SMO-Sequential Minimal Optimization.

### Description of CaspDB

The CaspDB database is publically available at http://caspdb.sanfordburnham.org for all users and no login or registering is required. It is a web-based, platform-independent database of predicted caspase cleavage sites in human proteins classified according to Uniprot.

#### User input

For user convenience there are two query options to retrieve cleavage site information of substrates. The CaspDB can be accessed by either Uniprot id or Uniprot name. By default, the retrieved information is for cleavage sites containing aspartic acid at P1 position, but the user can also add predictions with glutamic acid at P1 position as an option. Another way to retrieve cleavage site information of substrates is by using a motif search. For example a motif ‘DEVD-A/G/S/T’ has been observed to be preferred by caspase-3 in its target substrates. Using this motif search option all substrates containing a specified motif can be retrieved along with the prediction scores.

#### Results

All the results are provided in a user-friendly tabular form. The result page contains information about: a) the P1 position of cleavage sites with score values (in the range 0–1), predicted secondary structure and disorder characteristics, and cleavage prediction class (“yes” or “no”), b) the presence of a signal peptide, c) graphical and tabular descriptions of the domain structure of queried proteins according to Pfam annotations [28], d) the list of PTMs and SNPs, including disease annotation of the latter, e) a multiple sequence alignment with available orthologs.

The presence of signal peptide, as predicted by the SignalP v.4.0 program [29], indicates whether a protein is secreted. The domain annotation, which is included in the output page, is helpful in determining the inter- or intra-domain location of cleavage sites. All cleavage sites are arranged by default in descending order of score value. If a given substrate is experimentally annotated as a caspase substrate and reported in one of four known databases (MEROPS, Casbah, TopFIND 2.0 [30] and Degrabase [31]) then appropriate links to these databases are provided.

To check conservations of a substrate cleavage sites in other organisms, orthologous proteins from 11 organisms are retrieved and shown in tabular form. There is a Compare button to perform pair-wise comparison of cleavage sites between a given substrate and its orthologous proteins. Results are shown as a ClustalW pair-wise alignment along with cleavage sites tables for both the substrate and the ortholog, arranged next to each other in the form of look-up tables, which can be used for analysis of conservations of cleavage sites. Beneath this table, there is a button “Start Jalview” which can be used for displaying multiple sequence alignment of a substrate and all orthologous proteins. The output page also includes a list of SNPs and PTMs, with appropriate annotations, because both types of protein modification may affect caspase-mediated proteolysis.

### Case study and comparison

To further demonstrate the predictive power of CaspDB, we evaluated a list of published known caspase substrates (Table 2), which were not included in the RF model learning set. The CaspDB RF model was able to recognize all cleavage sites correctly with a high probability score. We describe three examples from Table 2 that are highlighted in bold. The first example is the Pyrin protein (MEFV_HUMAN, Uniprot ID: O15553), which is involved in innate immunity and the inflammatory response [32]. It interacts with several components of the inflammasome complex through which it recruits and activates caspase-1, leading to cleavage at the D330 position: TCVRD-SCSFP. CaspDB correctly predicted this cleavage site with a cleavage probability score of 0.735. This cleavage site was also recognized by the latest web-server Cascleave 2.0, which gives a low probability score of 0.546, on a scale ranging from 0 to 1. The second example is Cytosolic phospholipase A2 (PA24A_HUMAN, Uniprot ID: P47712), which is involved in the inflammatory response [33]. It has one experimentally determined caspase-8 cleavage site at D522: DDELD-AAVAD. CaspDB successfully predicted this cleavage site with a top score of 0.98, however Cascleave 2.0 scored this cleavage at 0.559. Another interesting example we evaluated with our model in which SNP (T300A) enhances caspase-3 cleavage efficiency is Autophagy-related protein 16-1 (A16L1_HUMAN, Uniprot ID: Q676U5) [13]. It has been observed that the T300A variant of A16L1 is sensitized to caspase-3-mediated cleavage, thereby revealing a functional connection between Crohn’s disease, caspase activation and autophagy. The experimentally known cleavage site of A16L1 is located at D299, within QDNVD-THPGS. For this protein our CaspDB model correctly predicts the cleavage site with a score of 0.965. We recalculated the score taking into account the SNP that leads to mutation T300A. This mutation increased the CaspDB score to 0.991 (QDNVD-AHPGS), in agreement with the results published by Murthy *et al.* [13] who observed that this SNP increases caspase cleavage efficiency. These results suggest that the CaspDB database can be used as useful tool for *in silico* cleavage site prediction. Inspection of Table 2 reveals that all of the cleavage sites are correctly and consistently scored highly by CaspDB.

**Table 2:** List of experimentally confirmed caspase substrates not included into the RF training set.

Uniprot_name	Caspase	P1	P5-P5’	Cascleave2.0 score	CaspDB score	Pubmed ID
ACTB_HUMAN	Casp-1	244	YELPD-GQVIT	0.512	0.939	9070648
**MEFV_HUMAN**	**Casp-1**	**330**	**TCVRD-SCSFP**	**0.546**	**0.735**	**18577712**
G3P_HUMAN	Casp-1	189	QKTVD-GPSGK	0.328	0.943	17959595
CING_HUMAN	Casp-3	173	LSSVD-SLINK	0.334	0.978	20058249
ASM_HUMAN	Casp-7	251	YSKCD-LPLRT	0.224	0.645	21157428
AT2B2_HUMAN	Casp-7	1117	VEEID-HAERE	0.282	0.912	12107825
COF1_HUMAN	Casp-6	17	KVFND-MKVRK	0.338	0.721	18487604
**PA24A_HUMAN**	**Casp-8**	**522**	**DDELD-AAVAD**	**0.559**	**0.98**	**9875225**
BAG3_HUMAN	Casp-3	215	RKEVD-SKPVS	0.473	0.984	20232307
**A16L1_HUMAN**	**Casp-3**	**299**	**QDNVD-THPGS**	**0.326**	**0.965**	**24553140**
			**QDNVD-AHPGS**	**n.d.**	**0.991**	
RUNX1_HUMAN	Casp-2	99	GDVPD-GTLVT	n.d.	0.961	24527765
MYD88_HUMAN	Casp-3	135	VAAVD-SSVPR	0.494	0.978	24363429
KDM4C_HUMAN	Casp-3	396	SDEVD-GAEVP	0.746	0.999	24952432
BMR1B_HUMAN	Casp-3	50	ICSTD-GYCFT	0.248	0.937	21368862
		120	RDFVD-GPIHH	0.448	0.992	
KKCC1_HUMAN	Casp-3	32	LEEAD-GGPEP	0.671	0.992	21368862
CSK_HUMAN	Casp-3	409	MDAPD-GCPPA	0.660	0.907	21368862
AKT2_HUMAN	Casp-3	121	EDPMD-YKCGS	0.714	0.943	21368862
KC1G1_HUMAN	Casp-3	343	SVHVD-SGASA	0.218	0.987	21368862
EF2 K_HUMAN	Casp-3	14	LEGVD-GGQSP	0.574	0.976	21368862
		430	HDHLD-NHRES	0.518	0.953	
MK12_HUMAN	Casp-3	46	CSAVD-GRTGA	0.519	0.834	21368862
MKNK2_HUMAN	Casp-3	32	LDQPD-HGDSD	0.429	0.931	21368862
		58	IDIPD-AKKRG	0.386	0.973	
PIM2_HUMAN	Casp-3	198	YTDFD-GTRVY	0.481	0.871	21368862
KPCI_HUMAN	Casp-3	6	PTQRD-SSTMS	0.474	0.987	21368862
TRIB3_HUMAN	Casp-3	338	QVVPD-GLGLD	0.467	0.985	21368862

Comparison of Cascleave 2.0 and CaspDB scores.

We also compared the predictive power of our CaspDB with Cascleave 2.0 using larger datasets. For this, we took known caspase-1 (114 cleavage sites from 100 substrates) and caspase-8 (57 cleavage sites from 38 substrates) substrates from the MEROPS database and calculated their cleavage site probability scores by both Cascleave 2.0 and CaspDB models.

**Figure 1:**
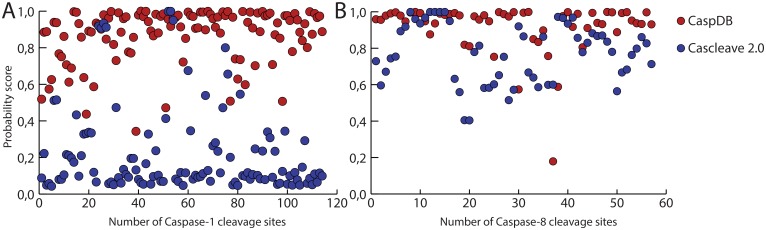
Comparision of CaspDB and Cascleave 2.0 scores. (A) Probability score comparison of caspase-1 cleavage sites. (B) Caspase-8 cleavage sites. CaspDB and Cascleave 2.0 scores are marked in red and black, respectively.

We found out that Cascleave 2.0 was able to score only 14 known cleavage sites of caspase-1 while CaspDB scores 111 out of 114 substrates above a threshold value of 0.5 (Figure 1A). For known cleavage sites of caspase-8, both Cascleave 2.0 and CaspDB predict cleavage positions correctly (Figure 1B). However, the CaspDB scores were higher than those obtained with Cascleave 2.0 in almost every case.

### SNPs and PTMs analysis

We performed an analysis on 520 experimentally known caspase substrates from the Casbah database for SNPs and PTMs in the vicinity of the scissile bond including P5-P4’ positions. We found 28 unique caspase substrates with SNPs in the cleavage motif. Among them, 10 substrates have SNPs that are known to be associated with disease. We found 198 unique caspase substrates, out of 520, with PTMs sites in the vicinity of cleavage site (Table S1, S2). These SNPs and PTMs may affect the efficiency of caspase cleavage efficiency. However, we shall utilize this list as a basis for future research.

## Conclusion

In summary, we provide a user-friendly database for retrieving information about potential caspase cleavage sites for all human proteins classified as “verified” according to Uniprot annotation. Our caspase cleavage prediction model works better in comparison to other available methods. As opposed to other databases and prediction tools, the CaspDB provides additional information that would be indispensable in the generation of new hypotheses and in the verification of new experimental findings concerning caspase-mediated cleavage of putative substrates. Information about cleavage in orthologous proteins is useful in assessing conservation of the cleavage positions across species, and thus in assessing the confidence of the prediction. Integration of SNPs and PTMs information is very useful, since one can inspect how these effects may alter the proteolytic event. Overall, our database will complement on-going experimental efforts in identifying new caspase substrates and further our understanding of the biochemistry of caspase-mediated substrate cleavage. This knowledge will be helpful for resolving the larger role of these proteases and their targets in critical processes, such as apoptosis, necroptosis and inflammation. As more information about caspases and their substrates becomes available, we will update and improve the performance of our methodology.

## Materials and Methods

### Datasets

We collected and curated substrate datasets for human caspases from the Casbah and MerCASBA databases. All the annotated substrate cleavage sites in the datasets were experimentally determined and thus not all putative cleavage sites were considered. The Casbah database contains 520 unique human proteins that are caspase substrates with a total of 661 experimentally determined cleavage sites (Table S3).

Our training dataset was constructed from this positive dataset of 661 known cleavage sites and a negative (non-cleavage sites) dataset. The negative set was constructed in the following way: we used a sliding window approach and took two upstream (P3 and P2) and two downstream (P1’ and P2’) residues from experimentally verified cleavage sequences, and tentatively assigned them to the P1 position. Thus, we have 661 and 2637 sequences in the positive and negative datasets (Table S3), respectively, in a 1∶4 ratio, as required by machine learning methods to accurately train classifiers. All the sequences within the training set were unique. The predicted models were built using several classifiers, as outlined below.

### Training of the classifiers

We tested several classifiers from the Weka machine learning software suit [27] for training our dataset for predicting putative caspase cleavage sites in human proteins. We chose to test four different classifiers: Random Forest (RF), Naïve Bayes (NB), decision tree algorithm (J48), and Sequential Minimal Optimization (SMO). We randomly divided our total positive and negative datasets into training (70%) and test (30%) datasets. The test datasets were used for evaluating the performance of each classifier model.

In our prediction model we combined information related to amino acid sequence and the predicted structural features, including secondary structure and disorder, into input for the Weka program. In 2009 Timmer et al. already recognized the importance of these structural features in caspase substrate [34]. Information about the amino acid preferences at every P5-P3’ position was defined in the form of the position weight matrix (PWM), which was calculated from known cleavage motifs in caspase substrates. The PWM has been calculated as follows. First, all sequences were aligned along the cleavage site and the frequency of occurrence of each amino acid in every P5-P3’ position was calculated. Next, the frequency of occurrence of each amino acid at every position was normalized by the distribution of amino acids in the set of background sequences, as defined in the MerCASBA database. Thus, the PWM value for each amino acid i_AA_ at the j^th^ position was calculated as:
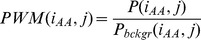



We used log_2_ values of each PWM (i_AA_, j) element of P5-P3’ position for further calculations. The input into the Weka program is composed of individual records (lines) for each positive and negative sequence from the dataset. For each sequence the input consists of appropriate values of the PWMs, for every amino acid at each position, along with, description of the secondary structure (α-helix: ‘H’, β-sheet: ‘E’, loop: ‘_’) and disorder (ordered: ‘.’ or disordered: ‘*’) for each residues at every P5-P3’ position. The secondary structure assignments and disorder regions were predicted using Jnet [35] and DISOPRED2 [36], respectively.

### Classifier evaluation

Each chosen classifier parameter (Table 3) was validated using a 10-fold cross validation. The number of true positives, false positives, true negatives and false negatives was counted and then the values of accuracy, precision, specificity, and Matthews Correlation Coefficient (MCC) and AUC (area under the receiver-operating characteristics (ROC) curve) parameters were calculated (Table 1). We also performed cost-benefit analysis using Weka, which determined the mean price/cost one should pay for “discarding” a very useful cleavage site and losing profit because of the wrong prediction taken by the classification model. Finally, all of these values were used for classifier evaluation. After classifier evaluation, we chose the best classifier for our cleavage prediction and then we bundled the test data back into training data to produce a set of input data to train the optimal select classifier for the actual prediction of caspase substrates in the entire human proteome (Table 1: RF_combined_set).

**Table 3:** Optimized parameter values for trained classifier.

Classifier	Parameters	Values
Random Forest (RF)	Maximum depth	Unlimited
	Number of Trees	1500
Naïve Bayes (NB)	Default	
J48	Confidence factor	0.25
	Number of folds	3
	Subtree raising	True
	Unpruned	False
	Use Laplace	False
SMO	Complexity	1
	Build logistic models	True
	Epsilon	1.0E-12
	Filter type	Normalize training data
	Kernel	RBF Kernel
	Gamma	0.01

### Prediction of caspase cleavage sites in human proteome

We downloaded all reviewed human proteins (20,266) from the Uniprot portal and then used CD-HIT [37] for eliminating redundant sequences. We used our best classifier model, Random Forest to predict the potential caspase cleavage sites in all human proteins.

For comparison of conservation of cleavage sites in other organisms, we extracted the protein sequences from the proteomes of additional 11 organism that are orthologous to human proteins (Table S4) from the OMG browser [38]. This retrieval of orthologous proteins was based on Uniprot id mapping. The information about experimentally known PTMs and SNPs in each human protein was obtained from curated dbPTM [39] and Humsavar [40], [41] databases, respectively. We also located the positions of cleavage sites with respect to protein domains according to information retrieved from the Pfam database [28].

### Web implementation

All predicted caspase cleavage sites in human proteins, mapped to their orthologous proteins, are stored online in the CaspDB database, available at: http://caspdb.sanfordburnham.org. CaspDB is currently configured on an Apache (CentOS) server hosted at the Sanford Burnham Medical Research Institute (SBMRI) with the application program Hypertext Preprocessor (PHP). It has been developed based on a combination of three layers. The underlying layer is the MySQL database system, a relational database management system that stores all the information on the putative cleavage sites of human proteins and their orthologs along with the Pfam domains, SNPs and PTMs in the back-end and provides the facility to link two or more tables in the database. The intermediate layer is an Apache-PHP application that receives the query from the user and connects to the database to fetch data from the upper layer, which comprises populated HTML and PHP pages, to the web browser client. The PHP and Java scripts are embedded in the HTML web pages and are used as application programs for integrating the back-end (MySQL database) with the web pages (HTML). Apache is used as the web server for building the interface between the web browser and the application programs. HTML and PHP have been used to build the web interface. ClustalW [42] and Jalview [43] were implemented to show the pairwise alignment and multiple sequence alignment, respectively.

There are no restrictions to use and access of CaspDB, which is publicly available at http://caspdb.sanfordburnham.org.

## Supporting Information

Table S1 **List of caspase substrates from Casbah database with SNPs in the vicinity of cleavage site.**
(XLS)

Table S2 **List of caspase substrates from Casbah database with PTMs in the vicinity of cleavage site.**
(XLSX)

Table S3 **List of training datasets.**
(TXT)

Table S4 **List of proteomes and their orthologs.**
(XLS)
